# Wnt/β-catenin signaling as an emerging potential key pharmacological target in cholangiocarcinoma

**DOI:** 10.1042/BSR20193353

**Published:** 2020-03-06

**Authors:** Guo-Feng Zhang, Ling Qiu, Shu-Li Yang, Jia-Cheng Wu, Tong-Jun Liu

**Affiliations:** 1 Department of Hepatobiliary and Pancreatic Surgery, The Second Hospital of Jilin University, Changchun 130041, China; 2 Department of Radiation Oncology, The Second Hospital of Jilin University, Changchun 130041, China; 3 Department of Obstetrics and Gynecology, The Second Hospital of Jilin University, Changchun 130041, China; 4 Department of Colorectal Surgery, The Second Hospital of Jilin University, Changchun 130041, China

**Keywords:** β-catenin, cholangiocarcinoma, macrophages, miRNA, Wnt

## Abstract

Cholangiocarcinoma (CCA) is a fatal malignant tumor of biliary epithelial cells involving intra- or extra-hepatic bile ducts. The prognosis of CCA is generally poor due to its diagnosis at the late stages. The currently employed chemotherapeutic agents do not increase the survival rate in patients with unresectable CCA. Accordingly, there is a need to identify new therapeutic agents for the effective management of intra- and extra-hepatic CCA. Clinical as well as preclinical studies have suggested the key role of the activation of Wnt/β-catenin signaling pathway in the induction and progression of CCA. There is an up-regulation of different Wnt ligands including Wnt2, Wnt3, Wnt5, Wnt7 and Wnt10 along with redistribution of β-catenin (more expression in the nucleus and lesser on the cell surface due to nuclear translocation of β-catenin) in different types of malignant biliary tumors. Apart from the role of this pathway in the induction and progression of CCA, this pathway is also involved in inducing multidrug resistance by inducing the expression of P-glycoprotein efflux pump on the cancer cells. These deleterious effects of Wnt/β-catenin signaling are mediated in association with other signaling pathways involving microRNAs (miRNAs), PI3K/AKT/PTEN/GSK-3β, retinoic acid receptors (RARs), dickkopf-1 (DKK1), protein kinase A regulatory subunit 1 α (PRKAR1A/PKAI), (SLAP), liver kinase B1 (LKB1) and CXCR4. The selective inhibitors of Wnt/β-catenin signaling may be potentially employed to overcome multidrug-resistant, fatal CCA. The present review discusses the role of Wnt/β-catenin along with its relation with other signaling pathways in the induction and progression of CCA.

## Introduction

Cholangiocarcinoma (CCA) is a fatal malignant tumor of biliary epithelial cells involving intra- or extra-hepatic bile ducts. The prognosis of CCA is generally poor due to its typical late diagnosis at the advanced stage, the ability to metastasize and relapse [1,2]. The incidence rate of intrahepatic cholangiocarcinoma (ICC) is increasing worldwide and it is the second most common form of primary liver cancer. The multifaceted risk factors involved in the development of CCA include the presence of liver diseases including biliary stones, infections, inflammatory bowel disease, pancreatitis, diabetes mellitus and obesity [3]. There is a need to identify new therapeutic agents for the effective management of intra- and extra-hepatic CCA.

Wnt/β-catenin signaling comprises Wnt ligands, which are 350–400 amino acids long lipid-modified glycoproteins and *CTNNB1* gene-encoded multifunctional β-catenin proteins [4]. In canonical Wnt/β-catenin signaling, the activation of Wnt leads to translocation of β-catenin into the nucleus, which in turn acts as a co-activator of transcription factors [5]. Among the different physiological and pathological functions performed by Wnt/β-catenin signaling, its role in the induction and progression of different types of cancers has been described [6]. Furthermore, the role of Wnt/β-catenin signaling in CCA has also been described. An up-regulation of the components of Wnt/β-catenin along with change in the expression pattern of β-catenin has been documented in the patients of CCA [7–11]. The present review discusses the role of Wnt/β-catenin along with its relation with other signaling pathways in the induction and progression of CCA.

## Current treatment modalities and future molecular targets

Surgery is the preferred treatment option for all types of CCA. Since most of the patients are diagnosed at the advanced stage, therefore, a minority of patients have the option of cure with the surgical removal of this aggressive [12]. Liver transplantation is another option for a small subset of patients with perihilar CCA [13] and ‘very early’ intrahepatic CCA [14]. For most of the patients with advanced-stage or unresectable CCA, the systemic chemotherapy comprising gemcitabine and cisplatin is mainly employed. However, the overall median survival with the currently available chemotherapy regimen is less than 1 year [15]. External-beam radiation therapy (EBRT) has also emerged as a valuable alternative for patients with localized, unresectable intrahepatic CCA [16]. The different new molecular targets that have been exploited in the preclinical and clinical trials for the management of CCA include fibroblast growth factor receptors [17], heat-shock protein 90 [18], tyrosine-kinase [19], ROS1 kinase fusion proteins [20], Akt/Erk signaling [21], SOX17, a transcription factor [22], Maternal Embryonic Leucine Zipper Kinase (MELK), a protooncogene [23] and mesothelin [24] etc. However, the development of drugs based on these molecular targets is still in the different stages of preclinical or clinical trials.

## Differential changes in the immunoexpression of β-catenin in CCA patients/cells

There have been a number of studies showing a decrease in the expression of β-catenin on the plasma membrane with a corresponding increase in the expression in the nucleus [25,26]. It has been shown that β-catenin is mainly expressed on the plasma membrane of the normal hepatocytes and bile ducts, and its nuclear expression is negligible. However, there is an increase in the nuclear expressions of β-catenin in hepatocellular carcinomas and CCAs. Moreover, the nuclear expression is relatively more common in high-grade carcinomas in comparison with low-grade carcinomas [27]. Immunohistochemical studies performed on the tissues isolated from 44 CCA patients revealed the significant decrease in the plasma membranous expression of β-catenin, which was correlated with tumors of high grade, vascular invasion and metastasis [28] (Table 1). Another study has documented the decrease in the expression of β-catenin in combined hepatocellular carcinoma and CCA (*n*=29), which was again correlated to the high grade of tumor and metastasis [29]. The study by Settakorn et al. [30] also elucidated the decrease in the membranous expression of β catenin with its corresponding increase in the nuclear expression in the surgically resected tissue from intrahepatic CCA patients (*n*=31). Accordingly, it is suggested that reduced immunoexpression of β-catenin on the plasma membrane and its overexpression in the nucleus may be used as prognostic markers in cholangiocarcinogenesis [30].

**Table 1:** Summarized details showing the role of Wnt/β-catenin signaling in the pathogenesis of different types of CCA in patients

S. No	Cancer type	Signaling pathway	Methodology	References
1.	CCA	Decrease in plasma membranous expression of β-catenin	Immunohistochemistry	Ashida et al., 1998
2.	Hepatocellular carcinoma and CCA	Decrease in plasma membranous expression of β-catenin	Immunohistochemistry	Asayama et al., 2002
3.	Intrahepatic CCA	Decrease in immunoexpression of β-catenin on plasma membrane and overexpression in nucleus	Immunohistochemistry	Settakorn et al., 2005; Tokumoto et al., 2005
4.	Extra-hepatic CCA	A parallel increase in nuclear expression of EpICD and β-catenin	Immunohistochemistry	Jachin et al., 2014
5.	Intrahepatic CCA	Overexpression of non-membranous β-catenin, EMT changes, decrease in E-cadherin and increase in vimentin expression	Immunohistochemistry	Huang et al., 2014
6.	CCA	Increase in the mRNA expression of Wnt3a, Wnt5a, and Wnt7b	RT-PCR	Loilome et al., 2014
7.	Hilar CCA	Increase in expression of Wnt2, Wnt3 and nuclear β-catenin	Immunohistochemistry	Chen et al., 2016
8.	Intrahepatic CCA	Loss of Secreted Frizzled-Related Protein-1 (SFRP1) protein	A decrease in SFRP1 protein expression increases β-catenin and results in poor prognosis	Davaadorj et al., 2017
9.	Intrahepatic CCA	Increase in the expression of Wls	A direct correlation between increase in Wls and growth of tumor	Shi et al., 2018

Abbreviation: EpICD, epithelial cell intracellular domain.

The data from the study of 37 biliary tract specimens obtained from biliary tract patients have shown mutations in the β-catenin gene suggesting the role of this gene in the poor prognosis in these cancer patients [31]. Another study has shown the mutations in the β-catenin genes in the 24 surgically resected samples of intrahepatic CCA. Moreover, immunohistochemical analysis showed the positive staining of β-catenin in the nucleus suggesting the differential changes in the immunoexpression of β-catenin in these patients [32]. The study performed by Gu and Choi [33,34] also reported the down-regulation of β-catenin on the plasma membrane, which was directly correlated with poor histological differentiation in CCA patients. Accordingly, it was proposed that reduced expression of membranous β-catenin is associated with a decrease in tumor differentiation and an increase in tumor progression [33,34].

It has been proposed that intramembranous proteolysis of epithelial cell adhesion molecule (EpCAM) may release an epithelial cell intracellular domain (EpICD) into the cytoplasm, which subsequently translocates in the nucleus to form a complex with β-catenin. The data obtained from the surgical specimens of extra-hepatic CCA patients and CCA cells have shown a correlation between the nuclear expression of EpICD and β-catenin. An increase in the nuclear expression of EpICD and β-catenin was also significantly correlated with histologic grade of the tumor, increased tumor growth and proliferation. On the other hand, small interfering RNA (siRNA)-mediated inhibition of EpCAM decreased cellular proliferation, migration and invasion suggesting the importance of localization of EpICD and its interaction with β-catenin in the progression and invasion of ECC [35] (Table 1).

## Involvement of β-catenin in inducing malignancy in normal cells and increasing proliferation in cancer cells

It has been proposed that β-catenin is involved in inducing malignancy in normal cholangiocytes. The study by Dutta et al. [36] described that there is a direct cell-to-cell transfer of oncogenic proteins via an exosomal pathway to induce malignancy in cholangiocytes. It was shown that exposure of normal cholangiocytes to CCA-derived exosomes increases the nuclear expression of β-catenin and induces malignancy in normal cells [36] (Table 2). Apart from induction, β-catenin also participates in the proliferation of CCA. It has been shown that human umbilical cord-derived mesenchymal stem cells or their conditioned media significantly increases the cancer cells proliferation and metastatic potency of CCA cells by stimulating the Wnt activity and promoting the nuclear translocation of β-catenin [37]. Accordingly, it may be proposed that cancerous cells shed the exosomes containing the oncogenes, which are transferred to normal cholangiocytes to induce malignancy by increasing β-catenin activity.

**Table 2:** *In vitro* studies showing the role of Wnt/β-catenin signaling in CCA cells

S. No	Experiments	Results	Comments	References
1.	Exposure of normal cholangiocytes to CCA-derived exosomes	Increase in nuclear expression of β-catenin and induction of malignancy in normal cells	Cancer cells shed exosomes containing oncogenes, which increase β-catenin activity to induce malignancy in normal cholangiocytes	Dutta et al., 2015
2.	Exposure of cholangiocytes to epithelial growth factor	Increase in nuclear translocation of β-catenin and disruption of adherens junctions	Epithelial growth factor contributes to CCA by inducing EMT through β-catenin	Clapéron et al., 2014
3.	Knock out of endogenous Fascin in QBC939 cells	Up-regulation of β-catenin in the plasma membrane along with inhibition of EMT, cellular proliferation and invasion	Fascin promotes EMT of CCA cells by increasing the nuclear and decreasing the plasma membrane localization of β-catenin	Mao et al., 2016
4.	Treatment of multidrug resistant CCA cells with β-catenin siRNA	Attenuation of P-glycoprotein efflux pump and multidrug resistance	Up-regulation of β-catenin contributes to the increase in expression of P-glycoprotein	Shen et al., 2013
5.	Treatment of multidrug-resistant CCA cells with β-escin	Decrease in multidrug resistance, degradation of β-catenin and down-regulation of P-glycoprotein	β-catenin participates in drug resistance by increasing the expression of P-glycoprotein	Huang et al., 2015
6.	Transfection of siRNAs targeting Wnt2 and β-catenin in FRH0201 cells (CCA cells)	Induction of apoptosis and suppression of cellular proliferation	Wnt2 and β-catenin are involved in proliferation of cancer cells	Zhang et al., 2013
7.	• Treatment of CCA cells with Wnt/β-catenin inhibitors • Treatment with uc.158- inhibitor	• Reduction in the expression of uc 158 • Reduction in the cellular proliferation	Activation of Wnt/β-catenin signaling increases the expression of uc.158- to increase cell proliferation	Carotenuto et al., 2017

Abbreviation: EMT, epithelial–mesenchymal transition.

## Role of Wnt/β-catenin in epithelial–mesenchymal transition in CCA and development of multidrug resistance

In CCA, the features of epithelial–mesenchymal transition (EMT) have been identified and EMT phenotypes are associated with poor survival rate in these patients [34]. It is shown that epithelial growth factor (EGF) contributes to the progression of CCA through EMT induction and exposure of cholangiocytes to EGF was shown to disrupt the adherens junctions and promote nuclear translocation of β-catenin [38]. A study performed on the tumor tissues of 140 intrahepatic CCA patients revealed the characteristic EMT changes in these tissues, in terms of a decrease in E-cadherin and an increase in vimentin expression. The EMT changes were correlated with the lymphatic metastasis and poorer overall survival. Moreover, the overexpression of non-membranous β-catenin, a major regulator of the EMT, was also identified in these patients [39]. The study by Mao et al. [40] described that fascin promotes EMT of CCA cells by increasing the nuclear and decreasing the plasma membrane localization of β-catenin. In QBC939 cells, knockout of endogenous Fascin expression by short hairpin RNA (shRNA) inhibited EMT, cellular proliferation and invasion, and tumor volume. Moreover, knockdown of Fascin up-regulated the expression of β-catenin on the plasma membrane fraction (a relative increase) and down-regulated the nuclear localization of β-catenin in the QBC939 cells. It suggests that restoration of β-catenin on the surface of the cell along with a decrease in its localization in the nucleus may prevent EMT and cellular proliferation in CCA [40].

The role of Wnt/β-catenin signaling in the development of multidrug resistance in CCA has also been described. In human multidrug-resistant CCA cells (QBC939/5-FU), the expression of P-glycoprotein and β-catenin was up-regulated. Inhibition of the Wnt/β-catenin signaling pathway by β-catenin siRNA overcame the multidrug resistance by decreasing the expression of the P-glycoprotein efflux pump [41]. In another study, the administration of β-escin was shown to abolish multidrug resistance in CCA cells by inducing degradation of β-catenin and down-regulating the expression of P-glycoprotein [42] again suggesting the importance of Wnt/β-catenin signaling in inducing drug resistance in CCA (Table 2).

## Increase in the expression of Wnt ligands in malignant biliary tumors

The role of different Wnt ligands in the development and progression of CCA has been described [9]. Based on the quantitative reverse transcription-polymerase chain reaction (RT-PCR), the increase in the mRNA expression of Wnt3a, Wnt5a and Wnt7b has been shown in the CCA tissues in comparison with non-tumor tissues. Moreover, siRNA-mediated suppression of β-catenin was shown to inhibit the growth of CCA cells [43]. Zhang et al. [44] described the inhibition of Wnt2 and β-catenin by transfection of siRNAs targeting Wnt2 and β-catenin induced cell apoptosis and suppressed cell proliferation in CCA cells (FRH0201 cells). Using *Opisthorchis viverrini* (one of the major etiological factors in the pathogenesis of CCA in northeast, Thailand)-induced cholangiocarcinogenesis in a hamsters, an up-regulation of Wnt/β-catenin signaling with an increase in the expression of Wnt3, Wnt3a, Wnt5a, Wnt7b and nuclear β-catenin in CCA tissues has been described [45] (Table 3). The immunohistochemistry-based study reported the increase in the expression of Wnt2, Wnt3 and nuclear β-catenin in the resected tissues isolated from hilar CCA patients suggesting the usefulness of Wnt ligands as a potential biomarker of in these patients [46].

**Table 3:** The role of Wnt/β-catenin signaling in animal models of CCA

S. No	Model	Results	Comments	References
1.	*Opisthorchis viverrini-*induced cholangiocarcinogenesis in hamsters	Increase in the expression of Wnt3, Wnt3a, Wnt5a, Wnt7b and nuclear β-catenin	Wnt/β-catenin signaling is involved in cancer	Yothaisong et al., 2014
2.	Thioacetamide model of CCA	• Increase in expression of Wnt10a during the pre-cancerous stage • A direct correlation between Wnt7b and tumor growth • A decrease in tumor area with the treatment of Wnt inhibitors, ICG-001 and C-59	• Role of Wnt10 in induction of tumor • Role of Wnt7b in the progression of cancer	Boulter et al., 2015

The role of Transcribed-ultraconserved regions (T-UCR), non-coding RNAs, downstream of the Wnt/β-catenin pathway has also described in biliary cancers. It was shown that there is overexpression of T-UCR uc.158- in CCA cells with nuclear localization of β-catenin. However, the expression of uc 158 was reduced following treatment with Wnt/β-catenin inhibitors. Furthermore, co-transfection of uc.158- inhibitor prevented cellular proliferation [47]. Boulter et al. [44] described the critical role of Wnt7b and Wnt10a in the pathogenesis of cholangiocarcinogenesis. The authors showed a higher expression of Wnt7b and Wnt10a (Wnt ligands) in human CCA. Particularly, Wnt7b protein was found to be present throughout the tumor tissue. The authors also confirmed the role of these Wnt ligands in CCA in experimental models in rodents. In the chemical (thioacetamide) model of CCA, it was shown that the expression of Wnt10a was increased during the pre-cancerous stage. On the other hand, a direct correlation was reported between the expression of Wnt7b and tumor growth. It suggests that Wnt10 may be involved in the induction of tumors; while Wnt7b may be involved in the progression of cancer. The role of Wnt signaling was further affirmed by the observations showing that specific inhibitors of Wnt pathway, i.e., ICG-001 and C-59 reduced the number and area of tumor in experimental models [48].

Davaadorj et al. [49] described that there is a loss of Secreted Frizzled-Related Protein-1 (SFRP1) protein in intrahepatic CCA tumor tissues, which was directly correlated to poor prognosis and negatively correlated to β-catenin expression. In other words, a decrease in the SFRP1 protein expression led to an increase in β-catenin expression and resulted in poor prognosis [49] (Table 1). It has been identified that hypermethylation in the promoter region of the Wnt inhibitory factor 1 (WIF-1) promoter region is crucial in the progression of cholecystitis to gallbladder cancer. Since WIF-1 plays a crucial role in controlling the expression of Wnt, therefore, it may be proposed that WIF-1 promoter-mediated changes in the expression of Wnt are important in the malignant tumor formation in gallbladders [50]. It also signifies the role of epigenetics in the induction of malignancy in the gallbladder. A previous study also documented the increase in the nuclear translocation of β-catenin in intrahepatic CCA patients is not a result of mutations in the exon portion of β-catenin [51]. Accordingly, it may be proposed that a decrease in the SFRP1 protein expression and hypermethylation in the WIF-1 promoter region may increase the expression of Wnt ligands to enhance Wnt/ β-catenin in CCA.

## Role of Wntless in malignant biliary tract tumors

Wntless (Wls) are transmembrane proteins that function to transfer the palmitoylated Wnt proteins from the endoplasmic reticulum to the plasma membrane. Since these proteins function to increase cell proliferation, decrease apoptosis and promote cell survival, therefore, the increased expression of these proteins is associated with the development and progression of different types of cancers [52,53]. The role of Wls in intrahepatic CCA was delineated by the study of Shi et al., [54] in which an increase in the expression of Wls was detected in the intrahepatic CCa tissues (*n*=44) using immunohistochemistry. The authors reported that immunoreactive Wls were detected in normal cholangiocytes, but not in normal hepatocytes. Moreover, the intensity for immunoreactive Wls was directly correlated with the tumor stage, tumor-node-metastasis stage and lymphatic invasion. Accordingly, it may be suggested that the expression of Wls is positively correlated to tumor stage and lymphatic invasion and it may be employed as a potential marker to diagnose the tumor stage and metastasis.

## The interrelationship between Wnt/β-catenin with other signaling pathways

**Figure 1:**
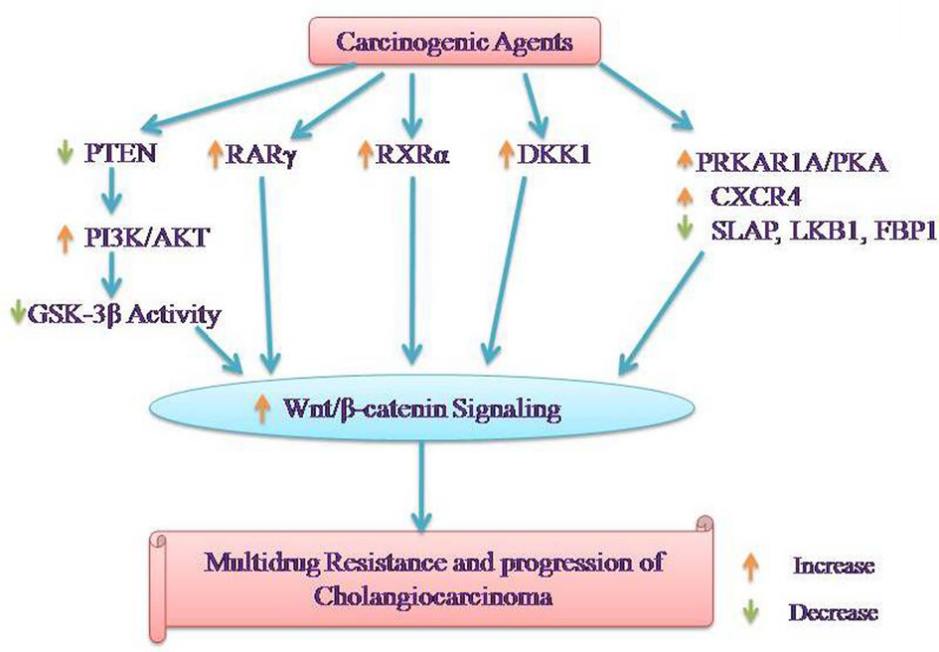
Representation of interrelationship between Wnt/β-catenin with other signaling pathways leading to the development of CCA

Scientists have explored the interrelationship between Wnt/β-catenin with other signaling pathways leading to the development of CCA (Figure 1).

### MicroRNAs

MicroRNAs (miRNAs) are approximately 20–25 nucleotide-long non-coding RNAs and these function to control the gene expression by binding to 3′-UTR of target mRNA [55]. There have been a number of studies documenting the key role of miRNA in different cancers [56]. Moreover, interplay between miRNA and Wnt/β-catenin signaling in regulating EMT in different cancers has also been reported [57]. The close interactions between different miRNAs and β-catenin in the pathogenesis of CCA have also been documented. The research data have revealed an increase in the expression of miR-26a in human CCA tissues and cells in comparison with non-cancerous biliary epithelial cells. Further, it was revealed that the activation of miR-26a led to a reduction in the GSK-3β activity followed by activation of β-catenin signaling. Inhibition of miR26a attenuated the growth rate of the tumor and prevented the activation of β-catenin. Conversely, depletion of β-catenin prevented miR-26a-induced tumor growth suggesting that miR-26a promotes CCA growth by inhibition of GSK-3β and subsequent activation of β-catenin [58]. In another study, an increase in the expression of miR-221 was shown in the specimens of extra-hepatic CCA and CCA cells. Furthermore, increased expression of miR-221 was strongly associated with the metastasis and prognosis of extra-hepatic CCA patients. It was also shown that miR-221 enhances β-catenin signaling to induce EMT in extra-hepatic CCA. Moreover, β-catenin also resulted in activation of miR-221 through c-jun suggesting the formation of a positive-feedback loop in extra-hepatic CCA cells [59]. Zhang et al. [60] determined that the interaction between miRNA and Wnt/β-catenin is mediated through non-protein coding, prostate cancer-associated transcript 1 (PCAT1). Indeed, it was shown that gene silencing of PCAT1 attenuated the progression of extra-hepatic CCA by inhibiting Wnt/β-catenin signaling through miR-122 repression. Accordingly, it may be possible to state that overexpression of PCAT1 may increase the miR-122 activity to activate Wnt/β-catenin signaling, which may be manifested in the form of an increase in progression of ECC. The important role of another miR-191 has been identified in CCA as its expression was found to be increased in the CCA cells and patients. The knockdown of miR-191 was shown to trigger apoptosis and prevent tumor progression. Furthermore, it was suggested that SFRP1 was the direct target of miR-191 and increase in miR-191 was associated with a decrease in the levels of SFRP1 and activation of Wnt/β-catenin signaling. Administration of SFRP1 siRNA re-activated the Wnt/β-catenin signaling pathway and restored the colony formation ability of cancerous cells [61].

**Figure 2:**
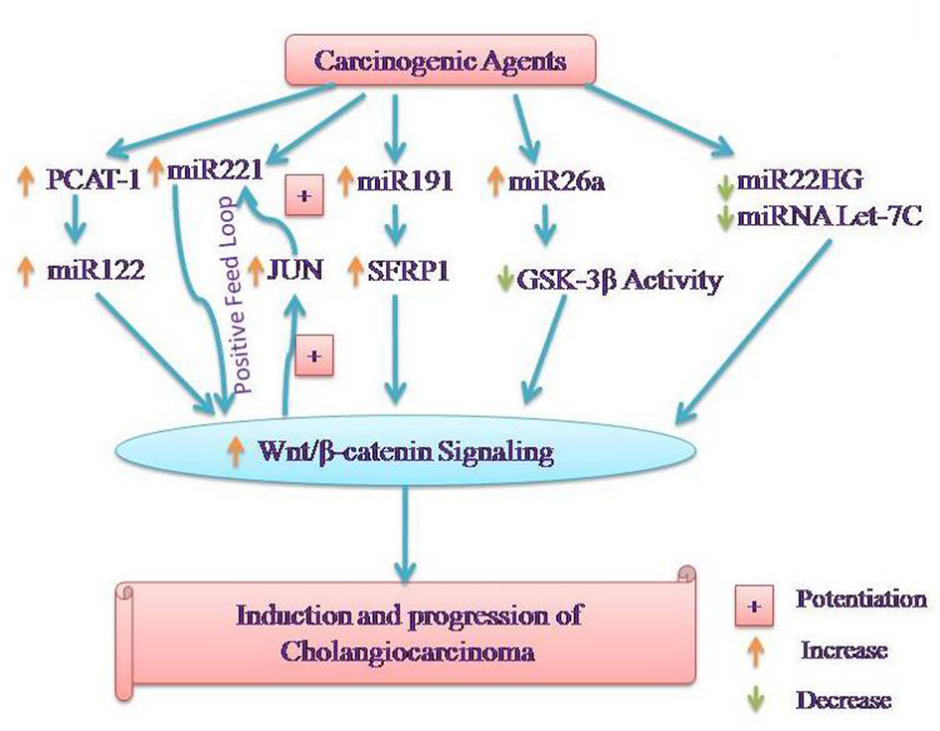
Representation of the interactions between miRNAs and Wnt/β-catenin signaling leading to the development and progression of CCA

In contrast with the above-described roles of miRNA in biliary cancers, the expression of MIR22HG was found to be significantly down-regulated in CCA tissues and cell lines. Moreover, MIR22HG negatively regulated the mRNA and protein expression of β-catenin. The overexpression of MIR22HG inhibited cell proliferation, migration and invasion in CCA along with a decrease in the expression of β-catenin [62]. The decrease in the expression of miRNA Let-7c has also been documented in CCA. Based on the *in vitro* and *in vivo* studies, it was reported that overexpression of miRNA Let-7c inhibits the tumorigenic properties of CCA by modulating β-catenin signaling pathway [63] (Figure 2).

### PI3K/AKT/PTEN/GSK-3β pathway

The PI3K-AKT-GSK-3β is a crucial intracellular signaling pathway and it serves to control a number of biological functions [64]. However, aberrant activation of this signaling cascade induces deleterious effects including the development of cancers, including CCA [65,66]. The studies have shown the interrelationships between PI3K/AKT/PTEN/GSK-3β and Wnt/β-catenin pathways in biliary tract cancers. Along with an increase in Wnt/β-catenin signaling, an up-regulation of the components of PI3K/AKT signaling including increase in expression of the p85α-regulatory and p110α-catalytic subunits of PI3K along with activation of AKT has been documented in Ov-induced cholangiocarcinogenesis in hamsters. Furthermore, the decrease in expression of PTEN, a negative regulator of PI3K/AKT, has also been reported suggesting the activation of PI3K/AKT signaling along with activation of the Wnt/β-catenin signaling in CCA [45]. There has been another study showing that the down-regulation of PTEN leads to activation of β-catenin to promote EMT in CCA cells [59]. Indeed, a survival analysis-based study showed that ICC patients with low PTEN have a worse clinical prognosis. Moreover, it was also shown that PTEN/PI3K/AKT and Wnt/β-catenin signaling is critical in enhancing EMT and ICC cell proliferation [67].

The interrelationship between GSK-3β/β-catenin has been described in the development of multidrug resistance in CCA and it is shown that Wnt3a activates GSK-3β/β-catenin signaling to up-regulate the expression of P-glycoprotein efflux pump on the tumor cells [42]. Another study has found that prostaglandin (PGE_2_) participates in the progression of CCA via EP3 receptors, which trigger PI3K/AKT/GSK-3β pathway to up-regulate the expression of β-catenin proteins [68]. The results of this study are supported by an earlier study documenting that cyclooxygenase-2-derived prostaglandin E_2_ leads to the activation of β-catenin in human CCA cells. Moreover in this study, administration of docosahexaenoic acid (DHA) was shown to dephosphorylate GSK-3β (increase in enzymatic activity), reduce the expression of β-catenin protein and attenuate the growth of tumor cells again. It suggests that the dephosphorylation of GSK-3β and increase in its enzymatic activity may induce protective effects by reducing the expression of β-catenin in CCA [69]. In another study, human umbilical cord-derived mesenchymal stem cells are shown to inhibit CCA cell growth in cell lines by decreasing the phosphorylation of AKT, GSK-3β and β-catenin. The association of a decrease in phosphorylation of GSK-3β (activation of its enzymatic activity), decrease in β-catenin and inhibition of CCA again suggests that the activation of GSK-3β may attenuate the growth of CCA by decreasing the expression of β-catenin. This contention was further supported by the findings showing that the treatment with GSK-3β inhibitor and AKT activator reversed the anticancer effects of stem cells and increased the expression of β-catenin [70].

### Retinoic acid receptors

Retinoic acid, a bioactive metabolite of vitamin A, is involved in regulating a large number of biological processes including cellular proliferation, differentiation and apoptosis. RARs (RARα, RARβ and RARγ), and RXRs are the retinoid receptors [71,72]. Studies have shown that the aberrant expression of RARγ is associated with the development of cancers including hepatocellular carcinoma [73]. Furthermore, the down-regulation of oncogenic RARγ down-regulates the Wnt/β-catenin signaling and abolishes the multidrug resistance in colorectal cancer [74]. The interrelationship between retinoic acid receptor γ (RARγ) and Wnt/β-catenin signaling has also been described in the development of chemoresistant bile duct carcinoma with a poor prognosis. In human CCA specimens, the overexpression of RARγ was documented and its overexpression was associated with poor differentiation, increase in metastasis and poor prognosis. At the molecular levels, it was revealed that the up-regulation of RARγ altered the distribution of β-catenin. In other words, an increase in nuclear translocation of β-catenin was documented in response to up-regulation of RARγ. It suggests that RARγ plays a key role in the proliferation, metastasis and development of chemoresistance in CCA through activation of Wnt/β-catenin signaling pathways [75]. The oncogenic role of another retinoid receptor, i.e. retinoid X receptor α (RXRα) has also been reported [76]. Moreover, the reciprocal relationship between RXRα and Wnt/β-catenin signaling in inducing the development of hepatocarcinoma has been described [77]. In CCA tissue, the overexpression and activation of RXRα has also been correlated with the activation of the Wnt/β-catenin signaling pathway. Moreover, the down-regulation of RXRα was shown to attenuate proliferation of CCA cells by suppressing Wnt/β-catenin signaling pathway [42].

### Dickkopf-1

The family of Dickkopf (DKK) comprises DKK1, DKK2, DKK3 and DKK4. Among the four members, the role of DKK1 is widely studied, including the progression of different cancers. In different types of cancers, the up-regulation of DKK1 has been positively correlated with the progression of cancers [78,79]. Moreover, it has been shown that it may participate in the progression of cancer by up-regulating the Wnt/β-catenin signaling [80]. The study of Shi et al. [81] described the possible relationship between DKK1 and β-catenin in HCCA cells. Based on the immunohistochemistry results, it was documented that there was a parallel increase in the expression of DKK1 in the human HCCA tissues and β-catenin expression. Along with it, this increase in the expression of both DKK1 and β-catenin was also correlated to the metastasis of cancer to the hilar lymph nodes. *In vitro* studies showed that the genetic depletion of DKK1 in the HCCA cells significantly reduces the expression of β-catenin along with inhibition of cellular proliferation and colony formation.

### Protein kinase A regulatory subunit 1 α pathway

The cAMP‐dependent protein kinase A (PKA) constitutes an important signaling molecule in a variety of cells and its enzymatic activity is regulated by two different regulatory domains, *viz.* PKA regulatory subunit 1 α (PRKAR1A/PKAI) and PKA regulatory subunit 2 β [82]. The importance of up-regulation of PRKAR1A in different cancers including in CCA has been emphasized [83]. Moreover, the role of PRKAR1A in the pathogenesis of CCA and its possible relation with the Wnt/β-catenin signaling pathway has been described. It was shown that the silencing of PRKAR1A and inhibition of PKA leads to inhibition of Wnt/β-catenin signaling, attenuates the growth and induces apoptosis in the CCA cells. It suggests that the overexpression of PRKAR1A/PKA may lead to the excessive activation of Wnt/β-catenin signaling, which in turn may participate in the progression of CCA [43].

### Other pathways

Studies have also suggested the interrelationship of Wnt/β-catenin with other signaling pathways in the induction and development of CCA. It has been shown that there is decrease in the expression of SRC-like adaptor protein (SLAP) [84] and liver kinase B1 (LKB1) [85] in ICC tissues and cells along with a decrease in fructose-1,6-bisphosphatase 1 (FBP1) in CCA cells [86]. Moreover, overexpression of SLAP in intrahepatic CCA cells [84] and FBP1 in CCA cells [86] is shown to induce apoptosis, decrease cellular proliferation and inhibit Wnt/β-catenin signaling. Moreover, an increase in the CXCR4 expression is shown to significantly lower the survival rate and CXCR4 knockdown is associated with the down-regulation of Wnt target genes and inhibition of progression of malignant tumor of the biliary tract [87].

## Conclusion

There is up-regulation of different Wnt ligands including Wnt2, Wnt3, Wnt5, Wnt7 and Wnt10 along with differential changes in the expression of β-catenin (more expression in the nucelus and less expression on the membrane) in different types of malignant biliary tumors. Accordingly, activation of Wnt/β-catenin pathway not only participates in the induction and progression of CCA, rather it may also induce multidrug resistance in these cancer cells. These deleterious effects of Wnt/β-catenin signaling are mediated in association other signaling pathway involving miRNAs, PI3K/AKT/PTEN/GSK-3β, RARs, DKK1, PRKAR1A/PKAI, SLAP, LKB1 and CXCR4. The employment of selective inhibitors of Wnt/β-catenin signaling may be potentially employed to overcome multidrug resistant, fatal CCA.

## Abbreviations

CCA: cholangiocarcinoma
DKK: Dickkopf
EGF: epithelial growth factor
EMT: epithelial–mesenchymal transition
EpCAM: epithelial cell adhesion molecule
EpICD: epithelial cell intracellular domain
FBP1: fructose-1,6-bisphosphatase 1
ICC: intrahepatic cholangiocarcinoma
LKB1: liver kinase B1
miRNA: microRNA
PCAT1: prostate cancer-associated transcript 1
PKA: protein kinase A
PRKAR1A/PKAI: PKA regulatory subunit 1 α
RAR: retinoic acid receptor
RXR: retinoid X receptor
SFRP1: secreted frizzled-related protein-1
siRNA: small interfering RNA
SLAP: SRC-like adaptor protein
T-UCR: Transcribed-ultraconserved region
WIF-1: Wnt inhibitory factor 1
Wls: Wntless
